# Personalized medicine in pancreatic cancer: Harnessing the potential of mRNA vaccines

**DOI:** 10.1016/j.jgeb.2025.100469

**Published:** 2025-02-17

**Authors:** Aariz Hussain, Areeba Fareed

**Affiliations:** Karachi Medical and Dental College, Pakistan

**Keywords:** Pancreatic cancer, mRNA vaccines, Treatment, Chemotherapy, Surgery, Personalized Medicine, Oncology, Autogene, Cevumeran

## Abstract

Pancreatic ductal adenocarcinoma (PDAC) is a highly lethal cancer with a five-year survival rate of just 7%. Its late diagnosis and limited treatment options contribute to poor outcomes. Immunotherapy has had little success due to PDAC’s dense and immunosuppressive tumor environment. Emerging mRNA vaccines, such as autogene cevumeran (BNT122), show promise in enhancing treatment. Preliminary trials have reported prolonged survival with minimal side effects. Despite this progress, the complexity of PDAC remains a significant challenge. Continued research is essential to fully realize the potential of mRNA-based therapies in combating this deadly cancer.

Dear Editor,

Pancreatic cancer, particularly pancreatic ductal adenocarcinoma (PDAC), is one of the deadliest cancers, with a five-year survival rate of just 7 % 1, 2. The global incidence of pancreatic cancer has alarmingly doubled over the past two decades, making it a significant contributor to cancer-related mortality. Despite advances in cancer treatment, the prognosis for pancreatic cancer remains bleak, largely due to the challenges in early detection and the limited effectiveness of current therapies.

The stealthy progression of pancreatic cancer, often presenting with nonspecific symptoms like back pain, decreased appetite, and weight loss in its advanced stages, complicates timely diagnosis ^3^. Standard diagnostic tools include serological tests and imaging techniques like endoscopic ultrasound (EUS) and MRI, but these are often employed too late in the disease's course ^3^. For the small percentage of patients eligible for surgery, the median five-year survival rate post-resection is only 25 %, with high recurrence rates 4, 5. The majority of patients, particularly those with advanced or metastatic PDAC, are left with limited options, often confined to palliative care.

The advent of immunotherapy has revolutionized treatment for many cancers, but its impact on PDAC has been limited. The FOLFIRINOX protocol is a key treatment for metastatic PDAC, though it comes with significant toxicity 5, 6. Immunotherapy, while transformative in treating other cancers, has shown limited success in PDAC due to the tumor's dense stroma and immunosuppressive microenvironment, which impede drug delivery 7, 8.

A promising development in this regard is the exploration of mRNA vaccines as a personalized treatment for pancreatic cancer. These vaccines, which are designed to target specific tumor antigens, represent a groundbreaking approach in the fight against this formidable disease ^9^.

A phase I clinical trial is currently investigating the safety and efficacy of an mRNA-based vaccine, autogene cevumeran (BNT122), in combination with chemotherapy and PD-L1 blockade in patients with resected PDAC. Preliminary findings have shown encouraging results, with patients exhibiting vaccine-induced immune responses and prolonged recurrence-free survival ^10^.

Among the sixteen patients who underwent surgery and received PD-L1 inhibition, all participants tolerated the autogene cevumeran treatment well. Notably, only one patient experienced a grade three fever and hypertension attributed to the vaccine, with no other grade three or higher adverse events observed. Moreover, half of the patients exhibited de-novo neoantigen-specific T cell responses, correlating with significantly prolonged recurrence-free survival. While the median duration remains undetermined, it surpasses 18 months, in contrast to 13.4 months in patients lacking vaccine-induced immune responses. These findings underscore the promising potential of personalized mRNA vaccines in pancreatic cancer treatment ^10^.

However, the complexity of pancreatic cancer, characterized by its heterogeneity and resistance mechanisms, presents ongoing challenges to the efficacy of these vaccines 9, 11. Ongoing research, including trials targeting KRAS mutations, is crucial to overcoming these obstacles and realizing the full potential of mRNA-based therapies ^12^.

A clinical trial (NCT05013216) is underway to assess the efficacy of a GNAS and KRAS-targeting peptide vaccine in preventing pancreatic cancer in high-risk individuals, with results expected in 2026. ^12^.

While pancreatic cancer remains a challenging disease with high mortality rates, the development of personalized mRNA vaccines offers a beacon of hope.

Novel approaches like mRNA vaccines show promise for personalized vaccine development, as demonstrated by preliminary findings from a phase I clinical trial investigating autogene cevumeran. However, the complex heterogeneity of pancreatic cancer and therapeutically resistant cells pose significant challenges to vaccine efficacy.

Moreover, the exploration of prophylactic mRNA vaccines against pancreatic cancer is in early stages, requiring further research and evaluation of effectiveness. Targeting oncogenic mutations like KRAS and GNAS offers a potential avenue for preventing pancreatic cancer development, but ongoing clinical trials are necessary to determine prophylactic efficiency.

Continued research and clinical trials are essential to refine these therapies and explore their potential in both treatment and prevention. By understanding the tumor microenvironment and targeting specific mutations, we may finally make significant strides in improving outcomes for pancreatic cancer patients.

## Financial Support

The authors did not receive any financial contribution for this research.

## CRediT authorship contribution statement

**Aariz Hussain:** Writing – review & editing, Writing – original draft. **Areeba Fareed:** Writing – review & editing, Writing – original draft.

## Ethics Approval

ERB Approval was not required for this research.

## Declaration of competing interest

The authors declare that they have no known competing financial interests or personal relationships that could have appeared to influence the work reported in this paper.
